# Bis(μ-4,4;6,6-bis­(biphenyl-2,2′-diyldi­oxy)-2,2-bis­{2-[5-(pyridin-4-yl)-1,3,4-oxadiazol-2-yl]phen­oxy}cyclo­triphosphazene)di-μ-chlorido-bis­[chlorido­copper(II)]

**DOI:** 10.1107/S1600536812016285

**Published:** 2012-04-21

**Authors:** Xiang-Wen Wu, Xiao-Yan Wang, Jian-Ping Ma, Yu-Bin Dong

**Affiliations:** a College of Chemistry, Chemical Engineering and Materials Science, Key Laboratory of Molecular and Nano Probes, Engineering Research Center of Pesticide and Medicine Intermediate Clean Production, Ministry of Education, Shandong Provincial Key Laboratory of Clean Production of Fine Chemicals, Shandong Normal University, Jinan 250014, People’s Republic of China

## Abstract

In the crystal of the title compound, [Cu_2_Cl_4_(C_50_H_32_N_9_O_8_P_3_)_2_], the binuclear mol­ecule is located across an inversion center. Each Cu^2+^ cation is coordinated by two pyridine N atoms from symmetry-related 4,4;6,6-bis­(biphenyl-2,2′-diyldi­oxy)-2,2-bis­{2-[5-(pyridin-4-yl)-1,3,4-oxadiazol-2-yl]phen­oxy}cyclo­triphosphazene (*L*) ligands, a pair of bridging Cl^−^ anions and a terminal Cl^−^ anion, forming a distorted CuCl_3_N_2_ square-pyramidal geometry. Weak intra­molecular C—H⋯O and inter­molecular C—H⋯N inter­actions occur in the crystal.

## Related literature

For our inter­est in the coordination chemistry of bent organic ligands bridged by five-membered heterocycles such as oxadiazole and triazole, see: Dong *et al.* (2005 ▶, 2007 ▶). For the impact of different types of linkages as well as distinct coord­inating orientations on the structures of various coordin­­ation-driven supra­molecular compounds, see: Zhao *et al.* (2007 ▶). For bond lengths and angles in related structures, see: Ainscough *et al.* (2008 ▶); Du *et al.* (2010 ▶). Zhao *et al.* (2009 ▶). 
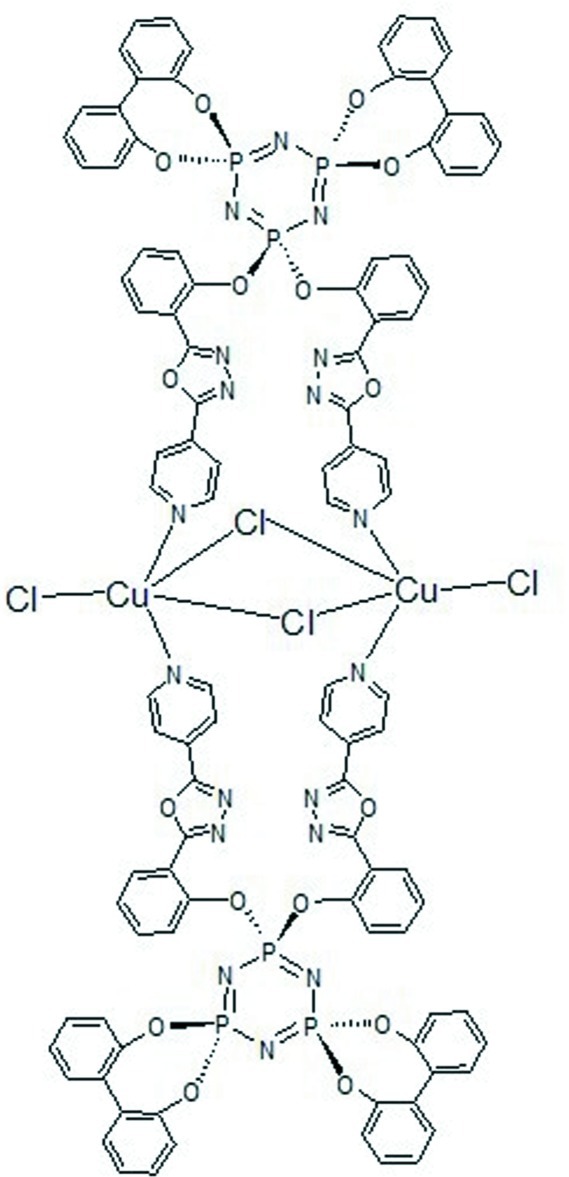


## Experimental

### 

#### Crystal data

[Cu_2_Cl_4_(C_50_H_32_N_9_O_8_P_3_)_2_]
*M* *_r_* = 2228.39
Triclinic, 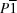

*a* = 10.554 (5) Å
*b* = 14.486 (7) Å
*c* = 15.551 (8) Å
α = 85.401 (7)°
β = 81.984 (7)°
γ = 89.844 (7)°
*V* = 2347 (2) Å^3^
*Z* = 1
Mo *K*α radiation
μ = 0.75 mm^−1^
*T* = 298 K
0.35 × 0.30 × 0.08 mm

#### Data collection

Bruker SMART 1000 CCD area-detector diffractometer
Absorption correction: multi-scan (*SADABS*; Bruker, 2001 ▶) *T*_min_ = 0.779, *T*_max_ = 0.942
12440 measured reflections
8593 independent reflections
6217 reflections with *I* > 2σ(*I*)
*R*_int_ = 0.027

#### Refinement

*R*[*F* ^2^ > 2σ(*F* ^2^)] = 0.056
*wR*(*F* ^2^) = 0.139
*S* = 1.02
8593 reflections
658 parameters
H-atom parameters constrained
Δρ_max_ = 0.51 e Å^−3^
Δρ_min_ = −0.48 e Å^−3^

### 

Data collection: *SMART* (Bruker, 2007 ▶); cell refinement: *SAINT* (Bruker, 2007 ▶); data reduction: *SAINT*; program(s) used to solve structure: *SHELXTL* (Sheldrick, 2008 ▶); program(s) used to refine structure: *SHELXTL*; molecular graphics: *SHELXTL*; software used to prepare material for publication: *SHELXTL*.

## Supplementary Material

Crystal structure: contains datablock(s) I, global. DOI: 10.1107/S1600536812016285/xu5503sup1.cif

Structure factors: contains datablock(s) I. DOI: 10.1107/S1600536812016285/xu5503Isup2.hkl

Additional supplementary materials:  crystallographic information; 3D view; checkCIF report

## Figures and Tables

**Table 1:** Hydrogen-bond geometry (Å, °)

*D*—H⋯*A*	*D*—H	H⋯*A*	*D*⋯*A*	*D*—H⋯*A*
C26—H26⋯O3	0.93	2.38	3.271 (5)	160
C36—H36⋯N5^i^	0.93	2.51	3.396 (6)	159

Symmetry code: (i) 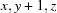.

**Fig. 1.**
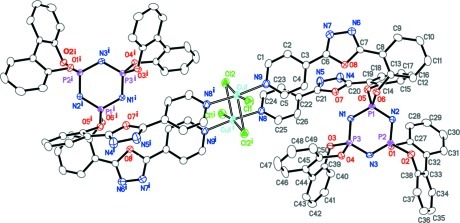
A view of CuII compound,showing the coordination around CuII. Displacement ellipsoids are drawn at the 30% probability level and H atoms have been omitted for clarity. [symmetry code: (i) -x, -y, -z + 1].

**Fig. 2.**
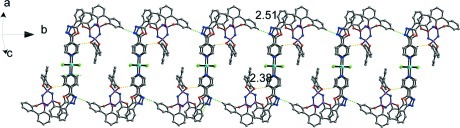
A diagram showing weak intermolecular C–H..N interaction.

## supplementary crystallographic information

### Comment

In our research, we have a longstanding interest in the coordination chemistry of bent organic ligands bridged by the five-membered heterocycles such as oxadiazole (Dong *et al.*, 2005) and triazole (Dong *et al.*, 2007), especially in how different types of linkages as well as distinct coordinating orientations impact the structures of various coordination-driven supramolecular compounds (Zhao *et al.*, 2007). In this contribution, we present a new ligand containing 1,3,4-oxadiazole and cyclotriphosphazene units, namely, bis(2,2'-dioxybiphenyl)bis(2-phenoxy-5-(4-pyridyl)-1,3,4-oxadiazole) cyclotriphosphazene (L) and the title compound, [CuCl_2_(C_50_H_32_O_8_N_9_P_3_)]_2_ based on it (Scheme 1).

In the crystal of the title compound, [CuCl_2_(C_50_H_32_O_8_N_9_P_3_)]_2_, the binuclear molecule is located across an inversion center (Figure 1). The Cu^II^ cation is N,N'-chelated by a bis(2,2'-dioxybiphenyl)bis(2-phenoxy-5-(4-pyridyl)-1,3,4-oxadiazole) cyclotriphosphazene (L) ligand, and a pair of bridging Cl^-^ anions and a terminal Cl^-^ anion further coordinate to the Cu^II^ cation to complete the distorted CuCl_3_N_2_ square-pyramidal geometry (Ainscough *et al.*, 2008). The N8^i^-Cu1-N9 angle is 174.68 (1)° and the dihedral angle between C1-C5/N9 and C21^i^-C25^i^/N8^i^is 21.272° (Zhao *et al.*, 2009) [symmetry code: i = -x, -y, 1-z]. The corresponding dihedral angles between the planes of the three rings in one 1,3,4-oxadiazole arm are 9.565° (between C1-C5/N9 and C6-C7-O8/N6-N7) and 5.322° (between C6-C7-O8/N6-N7 and C8-C13), and the corresponding dihedral angles of three rings in the other 1,3,4-oxadiazole arm are 30.941° (between C22-C26/N8 and C20-C21-O8/N4-N5) and 12.694° (between C20-C21-O8/N4-N5 and C14-C19). The Cu-N distances range from 2.021 (3) Å to 2.045 Å which are close to those found in {[Cu(4-bpo)(dca)(H_2_O)_2_](NO_3_)(H_2_O)}_n_ (4-bp = 2,5-bis(4-pyridyl)-1,3,4-oxadiazole, dca = N(CN)_2_^-^) (Du *et al.*, 2010). Moreover, the angles of Cl1-Cu1-Cl2 and Cl1-Cu1-Cl2^i^ are 167.54 (5)^°^ and 101.39 (5)°, respectively [symmetry code: i = -x, -y, 1-z]. The bridged Cu-Cl bond length (2.700 (6) Å) is longer than other Cu-Cl bonds (2.261 (9) Å and 2.300 (8) Å) (Ainscough *et al.*, 2008). Additionally, neighbouring Cu^II^ cations are connected by two bridged chlorine ions into Cu_2_Cl_2_ loops with a Cu···Cu separation 3.522 (1) Å.

In the solid state, binuclear molecules are linked together by weak intramolecular C—H···O and intermolecular C—H···N interactions into one-dimensional double-chains (Figure 2, Table 1).

### Experimental

Preparation of *L*:

The solution of 2-phenoxy-5-(4-pyridyl)-1,3,4-oxadiazole (0.50 g, 2.1 mmol) in 30 ml THF was added NaH(50.4 mg, 2.1 mmol), the mixture was stirred for 1 h at room temperature, then [N_3_P_3_(2,2'-dioxybiphenyl)_2_Cl_2_](0.574 g, 1 mmol) in 20 ml THF was added. The mixture was heated at reflux over 3 h, allowed to cool, the precipitation was separated by filtration and washed several times with water, then the residue was purified on a silica gel column using CH_2_Cl_2_:MeOH = 20:1 as the eluent to afford *L* as a white crystalline solid. IR(KBr pellet, cm^-1^): 1608(*m*), 1540(w), 1477(*m*), 1435(*m*), 1410(w),1276(*m*), 1233(*m*), 1175(*s*), 1090(*m*), 1033(*m*), 932(w), 890(*s*), 818(w), 785(*m*),750(*m*), 716(*m*), 701(*m*), 630(w), 608(*m*), 532(*m*), 409(w). ^1^H-NMR (300 MHz, 25°C, DMSO-d_6_,TMS, p.p.m.): 8.41–8.40(d, 2H, –C_5_H_4_N), 8.26–8.24(d, 1H, –C_6_H_4_O),8.02–7.84(d, 2H, –C_5_H_4_N), 7.76(d, 1H, –C_6_H_4_O),7.54(t, 1H, *p*-C_6_H_4_O), 7. 51–7.39(m, 3H, –C_6_H_4_O,-C_12_H_8_O_2_), 7.28–7.26(m, 4H, –C_12_H_8_O_2_),6.98–6.26(d, 3H, –C_6_H_4_O, –C_12_H_8_O_2_).Anal. Calcd. forC_50_H_32_N_9_O_8_P_3_: C, 61.29;H, 3.30; N, 12.86. Found: C, 61.33; H, 3.24; N, 12.89.

Preparation of Cu^II^ compound:

*L* (49 mg, 0.05 mmol) in CH_2_Cl_2_ (7 ml) and CuCl_2_.2H_2_O (9 mg, 0.05 mmol) in CH_3_OH (7 ml) were mixed. After about one week, green block-shaped crystals were obtained in 75% yield based on *L*.

### Refinement

H atoms attached to carbon were placed in geometrically idealized positions with C—H = 0.93 Å and refined using a riding model, with U_iso_(H) = 1.2U_eq_(C).

### Crystal data

**Table:**

[Cu_2_Cl_4_(C_50_H_32_N_9_O_8_P_3_)_2_]	*Z* = 1
*M**_r_* = 2228.39	*F*(000) = 1134
Triclinic, *P*1	*D*_x_ = 1.577 Mg m^−^^3^
Hall symbol: -P 1	Mo *K*α radiation, λ = 0.71073 Å
*a* = 10.554 (5) Å	Cell parameters from 2838 reflections
*b* = 14.486 (7) Å	θ = 2.2–24.7°
*c* = 15.551 (8) Å	µ = 0.75 mm^−^^1^
α = 85.401 (7)°	*T* = 298 K
β = 81.984 (7)°	Block, green
γ = 89.844 (7)°	0.35 × 0.30 × 0.08 mm
*V* = 2347 (2) Å^3^	

### Data collection

**Table:**

Bruker SMART 1000 CCD area-detector diffractometer	8593 independent reflections
Radiation source: fine-focus sealed tube	6217 reflections with *I* > 2σ(*I*)
Graphite monochromator	*R*_int_ = 0.027
φ and ω scans	θ_max_ = 25.5°, θ_min_ = 1.9°
Absorption correction: multi-scan (*SADABS*; Bruker, 2001)	*h* = −12→12
*T*_min_ = 0.779, *T*_max_ = 0.942	*k* = −17→13
12440 measured reflections	*l* = −18→18

### Refinement

**Table:**

Refinement on *F*^2^	Primary atom site location: structure-invariant direct methods
Least-squares matrix: full	Secondary atom site location: difference Fourier map
*R*[*F*^2^ > 2σ(*F*^2^)] = 0.056	Hydrogen site location: inferred from neighbouring sites
*wR*(*F*^2^) = 0.139	H-atom parameters constrained
*S* = 1.02	*w* = 1/[σ^2^(*F*_o_^2^) + (0.0629*P*)^2^ + 0.7991*P*] where *P* = (*F*_o_^2^ + 2*F*_c_^2^)/3
8593 reflections	(Δ/σ)_max_ = 0.001
658 parameters	Δρ_max_ = 0.51 e Å^−^^3^
0 restraints	Δρ_min_ = −0.48 e Å^−^^3^

### Special details

**Table:**

Geometry. All e.s.d.'s (except the e.s.d. in the dihedral angle between two l.s. planes) are estimated using the full covariance matrix. The cell e.s.d.'s are taken into account individually in the estimation of e.s.d.'s in distances, angles and torsion angles; correlations between e.s.d.'s in cell parameters are only used when they are defined by crystal symmetry. An approximate (isotropic) treatment of cell e.s.d.'s is used for estimating e.s.d.'s involving l.s. planes.
Refinement. Refinement of *F*^2^ against ALL reflections. The weighted *R*-factor *wR* and goodness of fit *S* are based on *F*^2^, conventional *R*-factors *R* are based on *F*, with *F* set to zero for negative *F*^2^. The threshold expression of *F*^2^ > σ(*F*^2^) is used only for calculating *R*-factors(gt) *etc*. and is not relevant to the choice of reflections for refinement. *R*-factors based on *F*^2^ are statistically about twice as large as those based on *F*, and *R*- factors based on ALL data will be even larger.

### Fractional atomic coordinates and isotropic or equivalent isotropic displacement parameters (Å^2^)

**Table:**

	*x*	*y*	*z*	*U*_iso_*/*U*_eq_	
C1	0.0712 (4)	−0.0313 (3)	0.2710 (3)	0.0457 (11)	
H1	0.0268	−0.0868	0.2868	0.055*	
C2	0.1569 (4)	−0.0249 (3)	0.1965 (3)	0.0453 (10)	
H2	0.1706	−0.0753	0.1629	0.054*	
C3	0.2228 (4)	0.0568 (3)	0.1715 (3)	0.0374 (9)	
C4	0.2035 (4)	0.1283 (3)	0.2261 (3)	0.0465 (11)	
H4	0.2500	0.1833	0.2130	0.056*	
C5	0.1149 (4)	0.1168 (3)	0.2994 (3)	0.0478 (11)	
H5	0.1009	0.1658	0.3347	0.057*	
C6	0.3034 (4)	0.0679 (3)	0.0879 (3)	0.0374 (9)	
C7	0.4205 (4)	0.1374 (3)	−0.0201 (2)	0.0362 (9)	
C8	0.4932 (4)	0.2111 (3)	−0.0736 (2)	0.0341 (9)	
C9	0.5458 (4)	0.1951 (3)	−0.1588 (3)	0.0462 (11)	
H9	0.5371	0.1369	−0.1787	0.055*	
C10	0.6096 (4)	0.2634 (3)	−0.2131 (3)	0.0518 (12)	
H10	0.6462	0.2505	−0.2687	0.062*	
C11	0.6206 (4)	0.3511 (3)	−0.1870 (3)	0.0467 (11)	
H11	0.6610	0.3979	−0.2254	0.056*	
C12	0.5710 (4)	0.3691 (3)	−0.1029 (3)	0.0379 (9)	
H12	0.5781	0.4281	−0.0845	0.045*	
C13	0.5108 (3)	0.2990 (3)	−0.0465 (2)	0.0317 (9)	
C14	0.7375 (3)	0.1807 (2)	0.0721 (2)	0.0295 (8)	
C15	0.8049 (4)	0.2253 (3)	−0.0015 (3)	0.0372 (9)	
H15	0.7672	0.2738	−0.0314	0.045*	
C16	0.9278 (4)	0.1988 (3)	−0.0314 (3)	0.0448 (10)	
H16	0.9738	0.2301	−0.0804	0.054*	
C17	0.9821 (4)	0.1253 (3)	0.0120 (3)	0.0453 (11)	
H17	1.0648	0.1068	−0.0077	0.054*	
C18	0.9139 (4)	0.0801 (3)	0.0839 (3)	0.0421 (10)	
H18	0.9515	0.0308	0.1128	0.051*	
C19	0.7891 (4)	0.1055 (3)	0.1157 (2)	0.0340 (9)	
C20	0.7238 (4)	0.0482 (3)	0.1890 (3)	0.0357 (9)	
C21	0.5882 (4)	−0.0021 (3)	0.2956 (3)	0.0413 (10)	
C22	0.4747 (4)	−0.0044 (3)	0.3607 (3)	0.0408 (10)	
C23	0.4200 (5)	−0.0890 (3)	0.3937 (3)	0.0535 (12)	
H23	0.4551	−0.1438	0.3742	0.064*	
C24	0.3138 (4)	−0.0910 (3)	0.4553 (3)	0.0530 (12)	
H24	0.2772	−0.1483	0.4759	0.064*	
C25	0.3143 (4)	0.0655 (3)	0.4566 (3)	0.0544 (12)	
H25	0.2792	0.1191	0.4788	0.065*	
C26	0.4198 (4)	0.0741 (3)	0.3937 (3)	0.0536 (12)	
H26	0.4539	0.1323	0.3737	0.064*	
C27	0.9432 (3)	0.4069 (2)	0.1586 (2)	0.0302 (8)	
C28	1.0052 (4)	0.3287 (3)	0.1306 (3)	0.0423 (10)	
H28	0.9665	0.2707	0.1433	0.051*	
C29	1.1253 (4)	0.3382 (3)	0.0836 (3)	0.0521 (12)	
H29	1.1684	0.2860	0.0641	0.062*	
C30	1.1819 (4)	0.4234 (3)	0.0652 (3)	0.0525 (12)	
H30	1.2630	0.4289	0.0329	0.063*	
C31	1.1200 (4)	0.5013 (3)	0.0940 (3)	0.0440 (10)	
H31	1.1596	0.5590	0.0809	0.053*	
C32	0.9980 (3)	0.4944 (3)	0.1429 (2)	0.0330 (9)	
C33	0.9357 (3)	0.5754 (2)	0.1815 (2)	0.0317 (8)	
C34	1.0032 (4)	0.6362 (3)	0.2232 (3)	0.0449 (10)	
H34	1.0895	0.6258	0.2264	0.054*	
C35	0.9449 (5)	0.7115 (3)	0.2601 (3)	0.0538 (12)	
H35	0.9917	0.7507	0.2886	0.065*	
C36	0.8176 (5)	0.7291 (3)	0.2551 (3)	0.0557 (13)	
H36	0.7788	0.7804	0.2796	0.067*	
C37	0.7478 (4)	0.6702 (3)	0.2135 (3)	0.0436 (10)	
H37	0.6624	0.6821	0.2086	0.052*	
C38	0.8072 (3)	0.5931 (2)	0.1793 (2)	0.0314 (8)	
C39	0.4887 (4)	0.3345 (2)	0.4399 (2)	0.0332 (9)	
C40	0.6046 (4)	0.3320 (3)	0.4705 (3)	0.0419 (10)	
H40	0.6766	0.3081	0.4386	0.050*	
C41	0.6109 (4)	0.3662 (3)	0.5505 (3)	0.0494 (11)	
H41	0.6879	0.3650	0.5732	0.059*	
C42	0.5037 (4)	0.4021 (3)	0.5964 (3)	0.0478 (11)	
H42	0.5083	0.4244	0.6504	0.057*	
C43	0.3902 (4)	0.4050 (3)	0.5632 (3)	0.0440 (10)	
H43	0.3188	0.4302	0.5948	0.053*	
C44	0.3791 (4)	0.3712 (3)	0.4831 (2)	0.0356 (9)	
C45	0.2570 (4)	0.3736 (3)	0.4468 (3)	0.0378 (9)	
C46	0.1428 (4)	0.3526 (4)	0.4998 (3)	0.0613 (13)	
H46	0.1432	0.3374	0.5590	0.074*	
C47	0.0272 (5)	0.3538 (4)	0.4663 (4)	0.0782 (17)	
H47	−0.0488	0.3409	0.5033	0.094*	
C48	0.0249 (4)	0.3740 (4)	0.3794 (4)	0.0704 (15)	
H48	−0.0522	0.3729	0.3570	0.084*	
C49	0.1367 (4)	0.3958 (3)	0.3248 (3)	0.0502 (11)	
H49	0.1359	0.4100	0.2655	0.060*	
C50	0.2493 (3)	0.3961 (3)	0.3593 (3)	0.0341 (9)	
Cl1	−0.18724 (11)	0.15324 (8)	0.35268 (7)	0.0535 (3)	
Cl2	−0.02705 (10)	−0.11280 (7)	0.46697 (7)	0.0485 (3)	
Cu1	−0.10057 (5)	0.02987 (3)	0.42097 (3)	0.04299 (16)	
N1	0.4580 (3)	0.3014 (2)	0.2006 (2)	0.0334 (7)	
N2	0.6647 (3)	0.3800 (2)	0.09859 (19)	0.0323 (7)	
N3	0.5960 (3)	0.4339 (2)	0.2594 (2)	0.0349 (8)	
N4	0.7652 (3)	−0.0316 (2)	0.2173 (3)	0.0536 (10)	
N5	0.6752 (4)	−0.0637 (2)	0.2876 (3)	0.0573 (10)	
N6	0.3977 (4)	0.0552 (3)	−0.0424 (3)	0.0599 (11)	
N7	0.3211 (4)	0.0102 (3)	0.0285 (3)	0.0599 (11)	
N8	0.2597 (3)	−0.0152 (2)	0.4874 (2)	0.0429 (8)	
N9	0.0475 (3)	0.0384 (2)	0.3230 (2)	0.0422 (8)	
O1	0.8252 (2)	0.39650 (16)	0.21153 (16)	0.0315 (6)	
O2	0.7351 (2)	0.53487 (16)	0.13627 (16)	0.0332 (6)	
O3	0.4819 (2)	0.29617 (16)	0.35980 (16)	0.0348 (6)	
O4	0.3585 (2)	0.42701 (16)	0.30209 (16)	0.0328 (6)	
O5	0.6110 (2)	0.20458 (16)	0.10054 (16)	0.0322 (6)	
O6	0.4665 (2)	0.31792 (16)	0.03865 (16)	0.0306 (6)	
O7	0.6120 (2)	0.07148 (17)	0.23493 (17)	0.0354 (6)	
O8	0.3621 (2)	0.15034 (17)	0.06086 (16)	0.0357 (6)	
P1	0.55400 (9)	0.30498 (6)	0.11381 (6)	0.0272 (2)	
P2	0.69841 (9)	0.43337 (6)	0.17686 (6)	0.0290 (2)	
P3	0.47897 (9)	0.36458 (6)	0.27569 (6)	0.0295 (2)	

### Atomic displacement parameters (Å^2^)

**Table:**

	*U*^11^	*U*^22^	*U*^33^	*U*^12^	*U*^13^	*U*^23^
C1	0.051 (3)	0.031 (2)	0.052 (3)	−0.0080 (19)	0.000 (2)	0.0047 (19)
C2	0.051 (3)	0.037 (2)	0.047 (3)	−0.005 (2)	0.001 (2)	−0.0062 (19)
C3	0.032 (2)	0.039 (2)	0.040 (2)	−0.0045 (17)	−0.0041 (19)	0.0002 (18)
C4	0.051 (3)	0.041 (2)	0.045 (3)	−0.018 (2)	0.001 (2)	−0.002 (2)
C5	0.052 (3)	0.038 (2)	0.051 (3)	−0.008 (2)	0.002 (2)	−0.009 (2)
C6	0.036 (2)	0.036 (2)	0.041 (2)	−0.0054 (18)	−0.0062 (19)	−0.0015 (18)
C7	0.035 (2)	0.043 (2)	0.031 (2)	−0.0004 (18)	−0.0019 (18)	−0.0093 (18)
C8	0.034 (2)	0.039 (2)	0.031 (2)	0.0005 (17)	−0.0085 (18)	−0.0037 (17)
C9	0.051 (3)	0.054 (3)	0.036 (3)	0.000 (2)	−0.007 (2)	−0.012 (2)
C10	0.049 (3)	0.072 (3)	0.031 (3)	−0.003 (2)	0.002 (2)	−0.003 (2)
C11	0.039 (2)	0.059 (3)	0.039 (3)	−0.009 (2)	−0.008 (2)	0.013 (2)
C12	0.037 (2)	0.041 (2)	0.037 (2)	−0.0014 (18)	−0.0115 (19)	0.0049 (18)
C13	0.0245 (19)	0.044 (2)	0.027 (2)	0.0016 (17)	−0.0081 (16)	0.0002 (17)
C14	0.029 (2)	0.030 (2)	0.031 (2)	0.0004 (16)	−0.0055 (17)	−0.0085 (16)
C15	0.039 (2)	0.038 (2)	0.035 (2)	0.0017 (18)	−0.0056 (19)	−0.0047 (18)
C16	0.047 (3)	0.046 (3)	0.039 (2)	−0.002 (2)	0.009 (2)	−0.010 (2)
C17	0.035 (2)	0.048 (3)	0.050 (3)	0.007 (2)	0.006 (2)	−0.009 (2)
C18	0.040 (2)	0.035 (2)	0.050 (3)	0.0127 (19)	−0.004 (2)	−0.0047 (19)
C19	0.035 (2)	0.033 (2)	0.034 (2)	0.0050 (17)	−0.0053 (18)	−0.0079 (17)
C20	0.031 (2)	0.034 (2)	0.043 (2)	0.0031 (17)	−0.0073 (19)	−0.0045 (18)
C21	0.043 (2)	0.035 (2)	0.045 (3)	−0.0003 (19)	−0.003 (2)	0.0003 (19)
C22	0.043 (2)	0.036 (2)	0.042 (3)	−0.0013 (19)	−0.004 (2)	0.0021 (18)
C23	0.065 (3)	0.034 (2)	0.057 (3)	0.001 (2)	0.005 (3)	0.000 (2)
C24	0.062 (3)	0.035 (2)	0.057 (3)	−0.005 (2)	0.009 (2)	0.002 (2)
C25	0.053 (3)	0.030 (2)	0.075 (4)	0.000 (2)	0.010 (3)	−0.004 (2)
C26	0.050 (3)	0.029 (2)	0.075 (3)	−0.006 (2)	0.011 (2)	0.005 (2)
C27	0.028 (2)	0.033 (2)	0.032 (2)	0.0014 (16)	−0.0115 (17)	−0.0037 (16)
C28	0.042 (2)	0.035 (2)	0.053 (3)	0.0091 (19)	−0.015 (2)	−0.013 (2)
C29	0.048 (3)	0.057 (3)	0.055 (3)	0.022 (2)	−0.011 (2)	−0.020 (2)
C30	0.032 (2)	0.075 (3)	0.048 (3)	0.015 (2)	0.005 (2)	−0.002 (2)
C31	0.033 (2)	0.050 (3)	0.047 (3)	0.0009 (19)	−0.004 (2)	0.004 (2)
C32	0.027 (2)	0.035 (2)	0.038 (2)	0.0000 (16)	−0.0070 (17)	−0.0032 (17)
C33	0.030 (2)	0.029 (2)	0.035 (2)	−0.0053 (16)	−0.0026 (17)	0.0024 (16)
C34	0.041 (2)	0.044 (3)	0.050 (3)	−0.013 (2)	−0.010 (2)	0.001 (2)
C35	0.073 (3)	0.035 (2)	0.055 (3)	−0.014 (2)	−0.011 (3)	−0.010 (2)
C36	0.083 (4)	0.029 (2)	0.052 (3)	0.006 (2)	0.002 (3)	−0.007 (2)
C37	0.047 (3)	0.032 (2)	0.050 (3)	0.0112 (19)	−0.004 (2)	−0.0016 (19)
C38	0.037 (2)	0.0268 (19)	0.030 (2)	−0.0067 (16)	−0.0049 (17)	0.0031 (16)
C39	0.042 (2)	0.026 (2)	0.032 (2)	0.0009 (17)	−0.0075 (18)	−0.0056 (16)
C40	0.043 (2)	0.041 (2)	0.042 (3)	0.0095 (19)	−0.010 (2)	−0.0032 (19)
C41	0.055 (3)	0.050 (3)	0.048 (3)	−0.003 (2)	−0.025 (2)	−0.001 (2)
C42	0.069 (3)	0.047 (3)	0.032 (2)	0.000 (2)	−0.019 (2)	−0.0078 (19)
C43	0.056 (3)	0.044 (2)	0.032 (2)	0.005 (2)	−0.003 (2)	−0.0062 (18)
C44	0.043 (2)	0.033 (2)	0.029 (2)	0.0008 (18)	−0.0039 (18)	−0.0014 (16)
C45	0.034 (2)	0.045 (2)	0.033 (2)	−0.0004 (18)	0.0013 (18)	−0.0040 (18)
C46	0.048 (3)	0.090 (4)	0.039 (3)	−0.002 (3)	0.010 (2)	0.010 (2)
C47	0.032 (3)	0.120 (5)	0.074 (4)	−0.003 (3)	0.008 (3)	0.020 (3)
C48	0.033 (3)	0.105 (4)	0.071 (4)	−0.008 (3)	−0.011 (3)	0.009 (3)
C49	0.039 (3)	0.067 (3)	0.045 (3)	−0.002 (2)	−0.010 (2)	0.001 (2)
C50	0.030 (2)	0.035 (2)	0.036 (2)	−0.0014 (17)	−0.0028 (18)	−0.0008 (17)
Cl1	0.0586 (7)	0.0497 (7)	0.0504 (7)	0.0054 (5)	−0.0059 (6)	0.0038 (5)
Cl2	0.0525 (6)	0.0362 (6)	0.0536 (7)	−0.0018 (5)	−0.0003 (5)	0.0037 (5)
Cu1	0.0438 (3)	0.0355 (3)	0.0460 (3)	−0.0034 (2)	0.0028 (2)	0.0034 (2)
N1	0.0297 (17)	0.0375 (18)	0.0327 (18)	−0.0091 (14)	0.0003 (14)	−0.0093 (14)
N2	0.0288 (17)	0.0365 (18)	0.0306 (18)	−0.0071 (14)	0.0017 (14)	−0.0060 (14)
N3	0.0347 (18)	0.0342 (18)	0.0373 (19)	−0.0070 (14)	−0.0036 (15)	−0.0132 (14)
N4	0.051 (2)	0.038 (2)	0.065 (3)	0.0100 (18)	0.008 (2)	0.0075 (18)
N5	0.059 (2)	0.040 (2)	0.064 (3)	0.0108 (19)	0.013 (2)	0.0109 (18)
N6	0.078 (3)	0.049 (2)	0.050 (2)	−0.018 (2)	0.012 (2)	−0.0161 (19)
N7	0.077 (3)	0.048 (2)	0.051 (3)	−0.020 (2)	0.011 (2)	−0.0135 (19)
N8	0.044 (2)	0.0320 (19)	0.049 (2)	−0.0043 (16)	0.0027 (17)	0.0017 (16)
N9	0.043 (2)	0.0317 (19)	0.049 (2)	−0.0015 (15)	0.0023 (17)	−0.0002 (16)
O1	0.0271 (13)	0.0271 (13)	0.0404 (16)	−0.0027 (10)	−0.0078 (12)	0.0023 (11)
O2	0.0288 (14)	0.0320 (14)	0.0402 (16)	−0.0006 (11)	−0.0099 (12)	−0.0021 (11)
O3	0.0455 (16)	0.0297 (14)	0.0301 (15)	0.0030 (12)	−0.0061 (12)	−0.0068 (11)
O4	0.0320 (14)	0.0348 (14)	0.0299 (15)	0.0003 (11)	0.0006 (12)	−0.0006 (11)
O5	0.0244 (13)	0.0305 (14)	0.0415 (16)	0.0024 (11)	−0.0046 (12)	−0.0022 (11)
O6	0.0260 (13)	0.0369 (14)	0.0301 (15)	0.0021 (11)	−0.0057 (11)	−0.0066 (11)
O7	0.0359 (15)	0.0322 (14)	0.0367 (16)	0.0044 (12)	−0.0026 (12)	0.0021 (12)
O8	0.0388 (15)	0.0338 (15)	0.0336 (16)	−0.0073 (12)	−0.0004 (12)	−0.0051 (12)
P1	0.0260 (5)	0.0304 (5)	0.0260 (5)	−0.0018 (4)	−0.0041 (4)	−0.0056 (4)
P2	0.0260 (5)	0.0290 (5)	0.0322 (6)	−0.0027 (4)	−0.0044 (4)	−0.0039 (4)
P3	0.0298 (5)	0.0305 (5)	0.0278 (5)	−0.0019 (4)	−0.0002 (4)	−0.0062 (4)

### Geometric parameters (Å, º)

**Table:**

C1—N9	1.344 (5)	C30—C31	1.377 (6)
C1—C2	1.364 (6)	C30—H30	0.9300
C1—H1	0.9300	C31—C32	1.400 (5)
C2—C3	1.376 (5)	C31—H31	0.9300
C2—H2	0.9300	C32—C33	1.472 (5)
C3—C4	1.387 (5)	C33—C38	1.384 (5)
C3—C6	1.449 (5)	C33—C34	1.389 (5)
C4—C5	1.370 (6)	C34—C35	1.377 (6)
C4—H4	0.9300	C34—H34	0.9300
C5—N9	1.339 (5)	C35—C36	1.378 (6)
C5—H5	0.9300	C35—H35	0.9300
C6—N7	1.289 (5)	C36—C37	1.384 (6)
C6—O8	1.356 (4)	C36—H36	0.9300
C7—N6	1.299 (5)	C37—C38	1.387 (5)
C7—O8	1.349 (4)	C37—H37	0.9300
C7—C8	1.450 (5)	C38—O2	1.405 (4)
C8—C13	1.394 (5)	C39—C40	1.372 (5)
C8—C9	1.401 (5)	C39—C44	1.382 (5)
C9—C10	1.363 (6)	C39—O3	1.414 (4)
C9—H9	0.9300	C40—C41	1.386 (5)
C10—C11	1.375 (6)	C40—H40	0.9300
C10—H10	0.9300	C41—C42	1.375 (6)
C11—C12	1.383 (6)	C41—H41	0.9300
C11—H11	0.9300	C42—C43	1.368 (6)
C12—C13	1.383 (5)	C42—H42	0.9300
C12—H12	0.9300	C43—C44	1.394 (5)
C13—O6	1.390 (4)	C43—H43	0.9300
C14—C15	1.374 (5)	C44—C45	1.477 (5)
C14—C19	1.387 (5)	C45—C46	1.382 (6)
C14—O5	1.399 (4)	C45—C50	1.387 (5)
C15—C16	1.381 (5)	C46—C47	1.391 (7)
C15—H15	0.9300	C46—H46	0.9300
C16—C17	1.380 (6)	C47—C48	1.363 (7)
C16—H16	0.9300	C47—H47	0.9300
C17—C18	1.363 (6)	C48—C49	1.376 (6)
C17—H17	0.9300	C48—H48	0.9300
C18—C19	1.400 (5)	C49—C50	1.371 (5)
C18—H18	0.9300	C49—H49	0.9300
C19—C20	1.448 (5)	C50—O4	1.405 (4)
C20—N4	1.300 (5)	Cl1—Cu1	2.2619 (14)
C20—O7	1.348 (4)	Cl2—Cu1	2.3008 (15)
C21—N5	1.279 (5)	Cl2—Cu1^i^	2.7006 (15)
C21—O7	1.366 (5)	Cu1—N9	2.021 (3)
C21—C22	1.455 (6)	Cu1—N8^i^	2.045 (4)
C22—C26	1.378 (5)	Cu1—Cl2^i^	2.7006 (15)
C22—C23	1.385 (6)	N1—P1	1.568 (3)
C23—C24	1.368 (6)	N1—P3	1.579 (3)
C23—H23	0.9300	N2—P2	1.575 (3)
C24—N8	1.337 (5)	N2—P1	1.578 (3)
C24—H24	0.9300	N3—P2	1.560 (3)
C25—N8	1.329 (5)	N3—P3	1.573 (3)
C25—C26	1.374 (6)	N4—N5	1.395 (5)
C25—H25	0.9300	N6—N7	1.391 (5)
C26—H26	0.9300	N8—Cu1^i^	2.045 (4)
C27—C28	1.377 (5)	O1—P2	1.587 (2)
C27—C32	1.384 (5)	O2—P2	1.580 (3)
C27—O1	1.395 (4)	O3—P3	1.580 (3)
C28—C29	1.373 (6)	O4—P3	1.584 (3)
C28—H28	0.9300	O5—P1	1.590 (3)
C29—C30	1.365 (6)	O6—P1	1.587 (2)
C29—H29	0.9300		
			
N9—C1—C2	123.5 (4)	C35—C34—H34	119.4
N9—C1—H1	118.2	C33—C34—H34	119.4
C2—C1—H1	118.2	C34—C35—C36	120.4 (4)
C1—C2—C3	119.3 (4)	C34—C35—H35	119.8
C1—C2—H2	120.4	C36—C35—H35	119.8
C3—C2—H2	120.4	C35—C36—C37	119.7 (4)
C2—C3—C4	118.0 (4)	C35—C36—H36	120.2
C2—C3—C6	119.7 (4)	C37—C36—H36	120.2
C4—C3—C6	122.1 (4)	C36—C37—C38	119.0 (4)
C5—C4—C3	119.1 (4)	C36—C37—H37	120.5
C5—C4—H4	120.5	C38—C37—H37	120.5
C3—C4—H4	120.5	C33—C38—C37	122.2 (3)
N9—C5—C4	123.2 (4)	C33—C38—O2	119.6 (3)
N9—C5—H5	118.4	C37—C38—O2	118.0 (3)
C4—C5—H5	118.4	C40—C39—C44	123.8 (4)
N7—C6—O8	111.7 (4)	C40—C39—O3	117.5 (3)
N7—C6—C3	128.3 (4)	C44—C39—O3	118.6 (3)
O8—C6—C3	119.8 (3)	C39—C40—C41	117.9 (4)
N6—C7—O8	111.7 (4)	C39—C40—H40	121.1
N6—C7—C8	126.8 (4)	C41—C40—H40	121.1
O8—C7—C8	121.4 (3)	C42—C41—C40	120.2 (4)
C13—C8—C9	117.2 (4)	C42—C41—H41	119.9
C13—C8—C7	124.0 (4)	C40—C41—H41	119.9
C9—C8—C7	118.8 (4)	C43—C42—C41	120.3 (4)
C10—C9—C8	121.1 (4)	C43—C42—H42	119.8
C10—C9—H9	119.5	C41—C42—H42	119.8
C8—C9—H9	119.5	C42—C43—C44	121.5 (4)
C9—C10—C11	121.0 (4)	C42—C43—H43	119.3
C9—C10—H10	119.5	C44—C43—H43	119.3
C11—C10—H10	119.5	C39—C44—C43	116.2 (4)
C10—C11—C12	119.4 (4)	C39—C44—C45	121.8 (3)
C10—C11—H11	120.3	C43—C44—C45	122.0 (4)
C12—C11—H11	120.3	C46—C45—C50	116.4 (4)
C13—C12—C11	119.7 (4)	C46—C45—C44	120.6 (4)
C13—C12—H12	120.1	C50—C45—C44	123.0 (4)
C11—C12—H12	120.1	C45—C46—C47	121.2 (5)
C12—C13—O6	118.6 (3)	C45—C46—H46	119.4
C12—C13—C8	121.4 (4)	C47—C46—H46	119.4
O6—C13—C8	120.0 (3)	C48—C47—C46	120.3 (5)
C15—C14—C19	120.9 (3)	C48—C47—H47	119.9
C15—C14—O5	120.8 (3)	C46—C47—H47	119.9
C19—C14—O5	118.0 (3)	C47—C48—C49	120.0 (4)
C14—C15—C16	120.6 (4)	C47—C48—H48	120.0
C14—C15—H15	119.7	C49—C48—H48	120.0
C16—C15—H15	119.7	C50—C49—C48	118.8 (4)
C17—C16—C15	119.5 (4)	C50—C49—H49	120.6
C17—C16—H16	120.3	C48—C49—H49	120.6
C15—C16—H16	120.3	C49—C50—C45	123.2 (4)
C18—C17—C16	119.7 (4)	C49—C50—O4	116.3 (4)
C18—C17—H17	120.2	C45—C50—O4	120.3 (3)
C16—C17—H17	120.2	Cu1—Cl2—Cu1^i^	89.14 (5)
C17—C18—C19	122.1 (4)	N9—Cu1—N8^i^	174.68 (13)
C17—C18—H18	119.0	N9—Cu1—Cl1	88.10 (10)
C19—C18—H18	119.0	N8^i^—Cu1—Cl1	90.93 (10)
C14—C19—C18	117.2 (4)	N9—Cu1—Cl2	88.67 (10)
C14—C19—C20	126.0 (3)	N8^i^—Cu1—Cl2	91.18 (10)
C18—C19—C20	116.8 (3)	Cl1—Cu1—Cl2	167.54 (5)
N4—C20—O7	112.5 (4)	N9—Cu1—Cl2^i^	94.56 (11)
N4—C20—C19	124.0 (4)	N8^i^—Cu1—Cl2^i^	90.77 (10)
O7—C20—C19	123.5 (3)	Cl1—Cu1—Cl2^i^	101.39 (5)
N5—C21—O7	112.5 (4)	Cl2—Cu1—Cl2^i^	90.86 (5)
N5—C21—C22	126.9 (4)	P1—N1—P3	120.96 (19)
O7—C21—C22	120.6 (3)	P2—N2—P1	120.2 (2)
C26—C22—C23	117.5 (4)	P2—N3—P3	121.78 (18)
C26—C22—C21	123.2 (4)	C20—N4—N5	105.9 (3)
C23—C22—C21	119.3 (4)	C21—N5—N4	106.6 (3)
C24—C23—C22	119.2 (4)	C7—N6—N7	106.3 (3)
C24—C23—H23	120.4	C6—N7—N6	106.7 (3)
C22—C23—H23	120.4	C25—N8—C24	116.7 (4)
N8—C24—C23	123.6 (4)	C25—N8—Cu1^i^	124.4 (3)
N8—C24—H24	118.2	C24—N8—Cu1^i^	118.8 (3)
C23—C24—H24	118.2	C5—N9—C1	116.7 (4)
N8—C25—C26	123.5 (4)	C5—N9—Cu1	122.2 (3)
N8—C25—H25	118.2	C1—N9—Cu1	120.5 (3)
C26—C25—H25	118.2	C27—O1—P2	120.5 (2)
C25—C26—C22	119.4 (4)	C38—O2—P2	120.3 (2)
C25—C26—H26	120.3	C39—O3—P3	118.2 (2)
C22—C26—H26	120.3	C50—O4—P3	124.2 (2)
C28—C27—C32	122.7 (4)	C14—O5—P1	128.2 (2)
C28—C27—O1	118.5 (3)	C13—O6—P1	121.9 (2)
C32—C27—O1	118.5 (3)	C20—O7—C21	102.5 (3)
C29—C28—C27	118.7 (4)	C7—O8—C6	103.6 (3)
C29—C28—H28	120.7	N1—P1—N2	118.73 (16)
C27—C28—H28	120.7	N1—P1—O6	104.80 (15)
C30—C29—C28	120.5 (4)	N2—P1—O6	110.24 (15)
C30—C29—H29	119.7	N1—P1—O5	109.29 (16)
C28—C29—H29	119.7	N2—P1—O5	110.41 (15)
C29—C30—C31	120.5 (4)	O6—P1—O5	101.93 (13)
C29—C30—H30	119.7	N3—P2—N2	117.71 (16)
C31—C30—H30	119.7	N3—P2—O2	111.60 (15)
C30—C31—C32	120.6 (4)	N2—P2—O2	105.09 (16)
C30—C31—H31	119.7	N3—P2—O1	105.30 (16)
C32—C31—H31	119.7	N2—P2—O1	112.52 (15)
C27—C32—C31	116.9 (3)	O2—P2—O1	103.87 (13)
C27—C32—C33	121.6 (3)	N3—P3—N1	118.07 (17)
C31—C32—C33	121.3 (3)	N3—P3—O3	112.23 (16)
C38—C33—C34	117.3 (3)	N1—P3—O3	105.70 (16)
C38—C33—C32	121.5 (3)	N3—P3—O4	104.77 (15)
C34—C33—C32	121.2 (3)	N1—P3—O4	112.16 (15)
C35—C34—C33	121.3 (4)	O3—P3—O4	103.00 (14)
			
N9—C1—C2—C3	−0.6 (7)	C46—C47—C48—C49	−1.9 (9)
C1—C2—C3—C4	3.1 (6)	C47—C48—C49—C50	0.5 (8)
C1—C2—C3—C6	−173.3 (4)	C48—C49—C50—C45	1.4 (7)
C2—C3—C4—C5	−3.7 (6)	C48—C49—C50—O4	−174.4 (4)
C6—C3—C4—C5	172.6 (4)	C46—C45—C50—C49	−1.8 (6)
C3—C4—C5—N9	1.9 (7)	C44—C45—C50—C49	178.0 (4)
C2—C3—C6—N7	2.3 (7)	C46—C45—C50—O4	173.8 (4)
C4—C3—C6—N7	−173.9 (4)	C44—C45—C50—O4	−6.3 (6)
C2—C3—C6—O8	175.9 (4)	Cu1^i^—Cl2—Cu1—N9	94.54 (11)
C4—C3—C6—O8	−0.3 (6)	Cu1^i^—Cl2—Cu1—N8^i^	−90.78 (11)
N6—C7—C8—C13	177.9 (4)	Cu1^i^—Cl2—Cu1—Cl1	169.5 (2)
O8—C7—C8—C13	1.2 (6)	Cu1^i^—Cl2—Cu1—Cl2^i^	0.0
N6—C7—C8—C9	0.2 (6)	O7—C20—N4—N5	−0.4 (5)
O8—C7—C8—C9	−176.5 (3)	C19—C20—N4—N5	−179.7 (4)
C13—C8—C9—C10	−1.0 (6)	O7—C21—N5—N4	0.0 (5)
C7—C8—C9—C10	176.8 (4)	C22—C21—N5—N4	−179.3 (4)
C8—C9—C10—C11	−2.2 (7)	C20—N4—N5—C21	0.2 (5)
C9—C10—C11—C12	2.9 (6)	O8—C7—N6—N7	−1.0 (5)
C10—C11—C12—C13	−0.2 (6)	C8—C7—N6—N7	−178.0 (4)
C11—C12—C13—O6	177.4 (3)	O8—C6—N7—N6	1.0 (5)
C11—C12—C13—C8	−3.2 (6)	C3—C6—N7—N6	175.0 (4)
C9—C8—C13—C12	3.7 (5)	C7—N6—N7—C6	0.0 (5)
C7—C8—C13—C12	−174.0 (3)	C26—C25—N8—C24	1.1 (7)
C9—C8—C13—O6	−176.8 (3)	C26—C25—N8—Cu1^i^	−178.7 (4)
C7—C8—C13—O6	5.5 (5)	C23—C24—N8—C25	−0.1 (7)
C19—C14—C15—C16	−2.9 (6)	C23—C24—N8—Cu1^i^	179.8 (4)
O5—C14—C15—C16	−177.2 (3)	C4—C5—N9—C1	0.7 (6)
C14—C15—C16—C17	1.5 (6)	C4—C5—N9—Cu1	−170.6 (3)
C15—C16—C17—C18	−0.2 (6)	C2—C1—N9—C5	−1.4 (6)
C16—C17—C18—C19	0.2 (6)	C2—C1—N9—Cu1	170.1 (3)
C15—C14—C19—C18	2.8 (5)	N8^i^—Cu1—N9—C5	133.5 (13)
O5—C14—C19—C18	177.3 (3)	Cl1—Cu1—N9—C5	54.0 (3)
C15—C14—C19—C20	−174.4 (3)	Cl2—Cu1—N9—C5	−138.0 (3)
O5—C14—C19—C20	0.1 (5)	Cl2^i^—Cu1—N9—C5	−47.2 (3)
C17—C18—C19—C14	−1.4 (6)	N8^i^—Cu1—N9—C1	−37.5 (15)
C17—C18—C19—C20	176.0 (4)	Cl1—Cu1—N9—C1	−117.0 (3)
C14—C19—C20—N4	165.9 (4)	Cl2—Cu1—N9—C1	51.0 (3)
C18—C19—C20—N4	−11.3 (6)	Cl2^i^—Cu1—N9—C1	141.8 (3)
C14—C19—C20—O7	−13.3 (6)	C28—C27—O1—P2	−113.1 (3)
C18—C19—C20—O7	169.6 (3)	C32—C27—O1—P2	72.5 (4)
N5—C21—C22—C26	147.9 (5)	C33—C38—O2—P2	71.7 (4)
O7—C21—C22—C26	−31.5 (6)	C37—C38—O2—P2	−112.3 (3)
N5—C21—C22—C23	−30.1 (7)	C40—C39—O3—P3	−103.8 (4)
O7—C21—C22—C23	150.6 (4)	C44—C39—O3—P3	77.0 (4)
C26—C22—C23—C24	1.5 (7)	C49—C50—O4—P3	−116.9 (3)
C21—C22—C23—C24	179.5 (4)	C45—C50—O4—P3	67.1 (4)
C22—C23—C24—N8	−1.3 (7)	C15—C14—O5—P1	−49.2 (4)
N8—C25—C26—C22	−0.9 (8)	C19—C14—O5—P1	136.3 (3)
C23—C22—C26—C25	−0.5 (7)	C12—C13—O6—P1	−89.1 (4)
C21—C22—C26—C25	−178.5 (4)	C8—C13—O6—P1	91.4 (4)
C32—C27—C28—C29	−1.4 (6)	N4—C20—O7—C21	0.5 (4)
O1—C27—C28—C29	−175.6 (3)	C19—C20—O7—C21	179.7 (3)
C27—C28—C29—C30	0.1 (6)	N5—C21—O7—C20	−0.3 (5)
C28—C29—C30—C31	0.5 (7)	C22—C21—O7—C20	179.1 (3)
C29—C30—C31—C32	0.2 (7)	N6—C7—O8—C6	1.5 (4)
C28—C27—C32—C31	1.9 (5)	C8—C7—O8—C6	178.7 (3)
O1—C27—C32—C31	176.1 (3)	N7—C6—O8—C7	−1.6 (4)
C28—C27—C32—C33	−173.3 (3)	C3—C6—O8—C7	−176.1 (3)
O1—C27—C32—C33	0.9 (5)	P3—N1—P1—N2	−6.0 (3)
C30—C31—C32—C27	−1.3 (6)	P3—N1—P1—O6	−129.6 (2)
C30—C31—C32—C33	173.9 (4)	P3—N1—P1—O5	121.8 (2)
C27—C32—C33—C38	−47.2 (5)	P2—N2—P1—N1	14.5 (3)
C31—C32—C33—C38	137.8 (4)	P2—N2—P1—O6	135.4 (2)
C27—C32—C33—C34	131.5 (4)	P2—N2—P1—O5	−112.8 (2)
C31—C32—C33—C34	−43.5 (6)	C13—O6—P1—N1	−162.0 (3)
C38—C33—C34—C35	−0.7 (6)	C13—O6—P1—N2	69.2 (3)
C32—C33—C34—C35	−179.4 (4)	C13—O6—P1—O5	−48.1 (3)
C33—C34—C35—C36	−1.1 (7)	C14—O5—P1—N1	−137.8 (3)
C34—C35—C36—C37	0.7 (7)	C14—O5—P1—N2	−5.5 (3)
C35—C36—C37—C38	1.4 (7)	C14—O5—P1—O6	111.7 (3)
C34—C33—C38—C37	2.8 (6)	P3—N3—P2—N2	15.3 (3)
C32—C33—C38—C37	−178.4 (4)	P3—N3—P2—O2	136.9 (2)
C34—C33—C38—O2	178.6 (3)	P3—N3—P2—O1	−111.0 (2)
C32—C33—C38—O2	−2.6 (5)	P1—N2—P2—N3	−18.9 (3)
C36—C37—C38—C33	−3.3 (6)	P1—N2—P2—O2	−143.86 (19)
C36—C37—C38—O2	−179.1 (4)	P1—N2—P2—O1	103.8 (2)
C44—C39—C40—C41	1.7 (6)	C38—O2—P2—N3	71.1 (3)
O3—C39—C40—C41	−177.6 (3)	C38—O2—P2—N2	−160.3 (3)
C39—C40—C41—C42	−0.6 (6)	C38—O2—P2—O1	−41.9 (3)
C40—C41—C42—C43	−0.6 (7)	C27—O1—P2—N3	−165.3 (2)
C41—C42—C43—C44	0.8 (7)	C27—O1—P2—N2	65.3 (3)
C40—C39—C44—C43	−1.5 (6)	C27—O1—P2—O2	−47.8 (3)
O3—C39—C44—C43	177.8 (3)	P2—N3—P3—N1	−7.1 (3)
C40—C39—C44—C45	179.1 (4)	P2—N3—P3—O3	116.2 (2)
O3—C39—C44—C45	−1.7 (6)	P2—N3—P3—O4	−132.7 (2)
C42—C43—C44—C39	0.2 (6)	P1—N1—P3—N3	2.3 (3)
C42—C43—C44—C45	179.7 (4)	P1—N1—P3—O3	−124.2 (2)
C39—C44—C45—C46	138.6 (4)	P1—N1—P3—O4	124.2 (2)
C43—C44—C45—C46	−40.9 (6)	C39—O3—P3—N3	55.3 (3)
C39—C44—C45—C50	−41.3 (6)	C39—O3—P3—N1	−174.7 (3)
C43—C44—C45—C50	139.3 (4)	C39—O3—P3—O4	−56.9 (3)
C50—C45—C46—C47	0.4 (7)	C50—O4—P3—N3	−148.7 (3)
C44—C45—C46—C47	−179.5 (5)	C50—O4—P3—N1	82.0 (3)
C45—C46—C47—C48	1.5 (9)	C50—O4—P3—O3	−31.2 (3)

Symmetry code: (i) −*x*, −*y*, −*z*+1.

### Hydrogen-bond geometry (Å, º)

**Table:**

*D*—H···*A*	*D*—H	H···*A*	*D*···*A*	*D*—H···*A*
C26—H26···O3	0.93	2.38	3.271 (5)	160
C36—H36···N5^ii^	0.93	2.51	3.396 (6)	159

Symmetry code: (ii) *x*, *y*+1, *z*.
